# *That’s not how the learning works* – the paradox of Reverse Innovation: a qualitative study

**DOI:** 10.1186/s12992-016-0175-7

**Published:** 2016-07-05

**Authors:** Matthew Harris, Emily Weisberger, Diana Silver, Viva Dadwal, James Macinko

**Affiliations:** Institute of Global Health Innovation, St Marys Hospital, Praed Street, London W2 INY and The School of Public Health, Imperial College London, Reynolds Building, St Dunstans Road, London, W6 8RP UK; Commonwealth Fund, 1 East 75th Street, New York, 10021 USA; Department of Nutrition, Food Studies and Public Health, New York University, 411 Lafayette Street, New York, 10003 USA; Johns Hopkins University Bloomberg School of Public Health, 615 N Wolfe St, Baltimore, MD 21205 USA; UCLA Fielding School of Public Health, Center for Health Sciences, 650 Charles E. Young Dr. South, Room 31-235B, Los Angeles, CA 90095-1772 USA

**Keywords:** Diffusion of innovation, Evidence based medicine, Developing countries

## Abstract

**Background:**

There are significant differences in the meaning and use of the term ‘Reverse Innovation’ between industry circles, where the term originated, and health policy circles where the term has gained traction. It is often conflated with other popularized terms such as Frugal Innovation, Co-development and Trickle-up Innovation. Compared to its use in the industrial sector, this conceptualization of Reverse Innovation describes a more complex, fragmented process, and one with no particular institution in charge. It follows that the way in which the term ‘Reverse Innovation’, specifically, is understood and used in the healthcare space is worthy of examination.

**Methods:**

Between September and December 2014, we conducted eleven in-depth face-to-face or telephone interviews with key informants from innovation, health and social policy circles, experts in international comparative policy research and leaders in the Reverse Innovation space in the United States. Interviews were open-ended with guiding probes into the barriers and enablers to Reverse Innovation in the US context, specifically also informants' experience and understanding of the term Reverse Innovation. Interviews were recorded, transcribed and analyzed thematically using the process of constant comparison.

**Results:**

We describe three main themes derived from the interviews. First, ‘Reverse Innovation,’ the term, has marketing currency to convince policy-makers that may be wary of learning from or adopting innovations from unexpected sources, in this case Low-Income Countries. Second, the term can have the opposite effect - by connoting frugality, or innovation arising from necessity as opposed to good leadership, the proposed innovation may be associated with poor quality, undermining potential translation into other contexts. Finally, the term ‘Reverse Innovation’ is a paradox – it breaks down preconceptions of the directionality of knowledge and learning, whilst simultaneously reinforcing it.

**Conclusions:**

We conclude that this term means different things to different people and should be used strategically, and with some caution, depending on the audience.

**Electronic supplementary material:**

The online version of this article (doi:10.1186/s12992-016-0175-7) contains supplementary material, which is available to authorized users.

## Background

Building on Christiansen’s notion of the ‘disruptive innovation’ [1], in the management literature the term ‘Reverse Innovation’ was first coined by Immelt [2] and then popularized by Govindarajan and Trimble [3]. Concerned that incumbent corporations would lose market share due to an inability to innovate, ‘Reverse Innovation’ was then the idea that disruptive innovations are best developed by first scaling them in low-income countries - lower barriers to entry, lower costs, and broader market base (bottom of the pyramid) – and then pursue market penetration back in the high-income country context [4]. The example of GE’s ultrasound machine and portable ECG machine are most-often cited: they were developed through local subsidiary organizations in India at a fraction of the cost and then introduced into the US market. In the management literature, the ‘Reverse Innovation’ process takes place within the boundaries, albeit often complex and diffuse ones, of the same multinational corporation [4]. Govindarajan and Trimble’s notion of Reverse Innovation is thus strategic and supply-led – developing a low-cost product to disrupt incumbents and develop new markets for growth.

In healthcare circles, however, ‘Reverse Innovation’ is taken to mean learning from, or diffusion of, the innovations that low-income countries have themselves developed and perhaps even scaled. This is a very different and far more complex process than that described in the management literature. Following Rogers [5], DePasse and Lee [6] suggest that it requires a ‘cross-over’ from Low-Income country early adopters to High-Income country innovators. They argue that Reverse Innovation requires ‘spannable social distances’ bridged by policymakers, entrepreneurs and health system leaders and utilizing diverse channels such as conferences, learning collaboratives and online resources [6]. From the high-income country (adopter) perspective it requires, first, knowledge of the innovation, then belief that it confers an advantage, and then persuasion of diverse local health system actors to consider it and adopt it. Compared to its use in the industrial sector, this conceptualization of Reverse Innovation describes a far more complex and fragmented process: one with no particular institution in charge, and blurring the lines between supply and demand. The actors driving this process may include innovation think tanks, health policy organizations and foundations and their work is to create demand – demand for the innovation and demand for local service providers interested (or persuaded) to pilot or adopt the innovation [7].

Reverse Innovation has become somewhat of a ‘movement’ – there is now a widespread array of organizations involved in this area, but also the increasing knowledge base that uses the term generated through academic, peer-reviewed publications as well as popular management literature. Following Brown and Zavestoski (2004) we define healthcare social movements as collective challenges to medical policy, public health policy and politics, belief systems, research and practice. Reverse Innovation fits this definition because of its challenge to the conceptualization that learning goes one way [8]. In healthcare circles, the claim to ‘newness’ of the ‘Reverse Innovation’ movement is curious. The notion that low-income countries have a lot to offer is not at all new. In the 1990s, Morgan and Rau [9] curated dozens of low-income country innovations worthy of adoption in high-income contexts in a process then known as ‘Global Learning for Health’. In 2004, the BMJ dedicated an entire volume to the learning that high-income countries could achieve from low-income ones, without one mention of the term ‘Reverse Innovation.’ In 2010, Lord Nigel Crisp, former Chief Executive of the NHS and Permanent Secretary of the UK Department of Health wrote that “It is increasingly recognized that innovation needs to be sourced globally’ but refers to this as a process of ‘co-development’ [10]. Clearly, one thing that is new about the ‘Reverse Innovation’ movement in health is its name [11].

Finally, there is an array of existing terms and often inter-changeable meanings that conjure different connotations or imagery to the contemporary umbrella term of ‘Reverse Innovation’. These include Frugal Innovation or Frugal Engineering [12], Innovation Blowback [13], Social Innovation [14], Co-development [10], as well as Trickle-up innovation, Bottom of Pyramid, Social entrepreneurship, and Leap-frogging. Although these terms and their meanings are related to innovative approaches, in and beyond healthcare, they nevertheless span geographic and thematic boundaries, having developed independently of the term ‘Reverse Innovation’. Other colloquial terms such as ‘jugaad’ (India), ‘hack’ (U.S.), and ‘*Système D’* (France) also refer to innovative fixes or simple work-arounds that solve complex issues. Additional file 1 provides more detail on some of these terms.

It follows that the way in which the term ‘Reverse Innovation’, specifically, is understood and used in the healthcare space is worthy of examination. This question constitutes one aspect of a broader deep dive into the barriers and challenges of the ‘Reverse Innovation’ process in the US that we undertook between September 2014 and June 2015. Here we present findings from interviews conducted with key informants and experts, each with experience of, or an interest in, Reverse Innovation or broader cross-country learning and diffusion of innovation. As part of interviews that explored in general the barriers and enablers to Reverse Innovation in the US, we asked our informants what they thought of the term ‘Reverse Innovation’, specifically focusing on their understanding, experience of and use of the term ‘Reverse Innovation’, and the connotations that it evokes, if any.

## Methods

### Sampling

Informants were selected purposefully from institutions and organizations known to have an interest in the Reverse Innovation space, and attention was given to ensure that there was representation from academic, non-profit foundation, health system management and innovation think tanks. To ensure an even wider representation, informants were also identified from both executive and managerial cadres and from those with experience or interest in international policy exchange in diverse disciplinary areas such as healthcare, as well as educational policy and social policy reform. Eleven participants were initially identified and all agreed to participate in the interviews which were conducted in-person or by telephone.

### Data collection

As exploratory research, with a focus on views and experiences, we used an open-ended interview style, loosely guided by some specific areas of interest, allowing for free-flowing conversation and examination of divergent themes [15]. These were to explore experiences of ‘Reverse Innovation’ in the US context, to identify the barriers and challenges from the informant’s point of view, and to enquire into their understanding of the ‘Reverse Innovation’ construct. Informants were first contacted by email and once the research was explained and agreement to participate obtained, interviews were arranged for dates, times and locations of their convenience. All the interviews were audio-recorded and verbatim transcripts were obtained from a commercial transcription company under strict confidentiality. Written informed consent was obtained from all informants. Participants had the right to withdraw their consent at any stage without giving a reason although none exercised this right. To facilitate voluntariness, potential participants received no inducement to participate. No identifying information was included in the transcripts and the informant list is kept securely apart from any data in a locked filing cabinet. Transcripts were checked and we removed any identifiable information or references immediately upon receipt by the research team and stored them in password-protected files. All interviews lasted between thirty minutes to an hour.

The research protocol was reviewed by the University Committee on Activities Involving Human Subject and deemed exempt from full ethical review (IRB# 14–10294). Interviews were all conducted by the same researcher (MH), during the period Sept-Nov 2014, and stopped once thematic saturation was reached.

### Analytical strategy

Two researchers (MH and EW) reviewed all the transcripts and using the first four transcripts independently indexed thematic categories using the process of constant comparison [16–19], indexing both vertically, within the same transcript, and horizontally, across the transcripts. These early code structures were then reviewed and revised for four further iterations, enabling the codes to be redefined, merged and retired until a final code structure had been obtained which was used to then code the remaining transcripts. Both researchers reviewed all coded transcripts, all of which had been coded independently using the final agreed code structure and any coding disagreements were resolved through consensus. The interviews generated over two hundred pages of verbatim transcript, which was manually coded. Coded data was organized into themes and sub-themes and the researchers identified patterns within the categorized data at higher levels of abstraction, developing explanatory concepts to link the themes where possible.

## Results

### Sales pitch – using the term Reverse Innovation to persuade

Several informants recognized that the process that Reverse Innovation refers to has been called many things but that this term, most of all, has a distinct, enduring quality. The claim to ‘newness’ in the Reverse innovation space obscures a different reality – learning from low-income countries is not new, but has been called by different names over the years:

‘[pilot project on a significant social policy reform adopted from Mexico] *was a combination of me and* [names a colleague] *at the World Bank, thinking about the challenges associated with north/south exchange of ideas and this kind of stuff. We didn’t call it….Reverse Innovation…..What did we call it? Yes…knowledge sharing.’* Professor of Applied Psychology (28^th^ October 2014)

The resurgence of interest in this area is inextricably linked to the name itself, and can in some senses be viewed as a successfully branded management strategy to effect organizational change and reignite interest in learning from low-income countries. Many informants considered the term Reverse Innovation to be part of a good ‘sales pitch’ because it uses positive, change language managers can understand. It is a popularized term, suggesting a well-established management strategy or evoking the sense that there is a body of evidence to sustain it, giving comfort to policy makers concerned with adopting a policy from a non-traditional, or non-familiar, context.

‘*I think it* [the term Reverse Innovation] *is a terrific sales pitch. When I go to an insurance regulator, it is much harder to say look, we have some problems here, they have this great thing in Mexico, let’s try to think about how that business model can work here. That is a much harder sales pitch than when I say hey, there’s this phenomenon called Reverse Innovation, and lots of people are talking about it, and its this widespread phenomenon…and this is a particular example of Reverse Innovation…I think it gives a lot of comfort to policy makers who are worried about doing something risky.’* Professor of Law (1^st^ October 2014)

‘*The other thing is I think a total preoccupation with innovation, you know, as a concept. I mean there’s just such a growth, a proliferation of….its probably the most overused terms in healthcare today….and if not in other sectors. It might be just generally in any business, innovation might be sort of an overused term overall, you know? And so the notion of innovation itself has risen to the surface and has…is…has kind of become in vogue and kind of the thing.’* VP Innovation think tank (10th October 2014)

### Reverse Innovation is associated with frugality and can be offensive

Although the term ‘Reverse Innovation’ seems to have a positive branding quality, the use of and interpretation of the term is not, as many noted, entirely without issue. Many informants recognized that by virtue of the Reverse Innovation process being associated with low-income contexts, it could create an immediate association that the innovation is cheaper than existing technologies. Whilst many of the innovations proposed within the Reverse Innovation space are indeed cheaper, and although reducing cost on its own can be an important goal in a health system, many informants noted that reduced cost can equate to reduced quality and this makes it harder to be convincing. It renders the term ‘Reverse Innovation’ somewhat counterproductive, damaging the perceived relative advantage of the innovation, evoking images of cheap solutions best kept in locations that need to use them.

Low-income countries such as Ethiopia and Pakistan have scaled a lay health worker cadre to national levels with well-described benefits for those contexts. One informant noted that the term ‘Reverse Innovation’, when associated with adoption of a similar model in the US, results in a somewhat negative reaction that serves to undermine the learning aspiration:

‘*Well, the term that I’ve often thought about is right-skilling, which is kind of deskilling, right, which is we want to have less expensive, less highly trained people doing certain tasks. That also sounds dangerous…They say wait a second, all this really is, you know, is going away from a shiny building into somebody’s garage, and that’s kind of dangerous when you think about the healthcare world where these policy makers job is to keep people safe.’* Professor of Law (1^st^ October 2014)

It is not the innovation per se that evokes a sense of ‘*going away from a shiny building into somebody’s garage*’ but awareness of the source of the innovation, implied immediately by the term ‘Reverse Innovation’. The term ‘Reverse Innovation’ invokes the image of low-income contexts from whence the innovation came. Whether accurate or not, a host of important inferences are subsequently made about the context and the innovation. For example, informants often noted that innovations worthy of examination from low-income contexts had been developed ‘out of necessity’ – as if to say, resource-poor contexts have to do more with less and therefore the systems that are developed are leaner:

‘*I think where it’s gotten traction is the idea that we really can learn from what’s happening in middle- and low-income countries because those are the places where the resource constraints are most severe, and where the needs are most urgent….we’re learning from places that have to be frugal by necessity and now we can learn from that’.* Executive Director, Innovation think tank (1^st^ October 2014)

This narrative is problematic because it asserts that cost pressures alone, and not good leadership for example, led somewhat organically to the low-cost/high-value innovation in the low-income country. It also implies that in the US there is no cost pressure at all, a burden shouldered only in low-income contexts. Many informants noted that the use of the term Reverse Innovation could therefore be considered as offensive, misplaced or derogatory in the innovator context:

‘*It’s offensive, it’s derogatory for the people that are coming up with the ideas and it creates already stereotypes and prejudices so I think it’s a terrible name….the politicians and leaders of these countries probably are not that excited about having their country branded as, you know, an unusual suspect for a good idea’* Manager, Innovation think tank (1^st^ October 2014)

### Reverse Innovation is a paradox

Our final observation from the interviews is that the term ‘Reverse Innovation’ is somewhat of a paradox. None of the informants specifically referred to the term ‘Reverse Innovation’ as being a paradox however reviewing the interpretations and uses of the term leads us to consider it being one. First of all, the term Reverse Innovation has stuck:

‘*I’ve seen other terms. I’ve seen bidirectional or multidirectional innovation, or generalized innovation, which somehow gets there….but none of these have the same kind of cultural cachet….or the same kind of penetration or regularity of use as Reverse Innovation. Reverse Innovation, for whatever reason, seems to have stuck, as you know, as the phrase that people recognize this kind of innovation by.’* VP Innovation think tank (10^th^ October 2014)

even though many do not like the term at all:

‘*Well I don’t like the term Reverse Innovation….I hate it.’* (Senior Manager, Innovation think tank (1^st^ October 2014)

‘*….none of us like the term at all. It’s something we’ve talked a lot about, both as a team but with, of course, our cooperators in this space, and* [fact that the term is very problematic] *comes up in our meetings.’* Manager, Innovation think tank (1^st^ October 2014)

The term readily perpetuates the same colonial world-view that it supposedly serves to break down:

‘*…the term assumes you’re taking ideas from countries either in transition or with nothing and taking what they’re doing in a frugal or low resource way and making it useful here because if they had the same means that we had you wouldn’t necessarily need to brand it that way…..you would just call it knowledge sharing or like stimulating idea flow or something, it would be totally flat….’* Senior Manager, Innovation think tank (1^st^ October 2014)

‘*….it* [the term Reverse Innovation] *assumes a world in which the flow of information is expected to go from high income to low income countries, and anything happening in the other direction would be reverse….I find that problematic.’* Manager, Innovation think tank (1^st^ October 2014)

‘*…like we were at the center of innovation all the time, most of it we export and give to you* [low-income countries] *and now we’re going to reverse it, it trickles back.’* Senior Manager, Innovation think tank (1^st^ October 2014)

‘*….I try and get away from this notion that there’s an us-and-them kind of concept, which I think is embedded in the notion of “Reverse Innovation”* ‘VP Innovation think tank (10^th^ October 2014)

This ‘colonial view’ is so deeply entrenched within the term ‘Reverse Innovation’, that one informant admitted to feelings of exploitation when drawing on the experiences of a low-income country. In the following excerpt, the informant describes a fairly convoluted, institutional approach to mitigate ‘exploiting’ the low-income country. There is a distinct sense that using knowledge, experience or evidence from a low-income context is not fair game, something needs to be given back:

‘*…..arguably, we could do the learning from these projects without ever making any grant, you know, without providing funding to these organizations. We could go and visit them and talk to them, sort of, extract that learning and then think about how to come back here and get it going in the United States, but, I think, we wanted to be conscious of not appearing like we were exploiting that, those organizations particularly in the Global South. So our grant funding is a bit transactional in some ways. That is, that, sort of, our support of their work allows us, I think, to feel like it’s a bit of an exchange, in a way that makes us feel like we’re not taking advantage of them but, sort of, respectfully treating them like the peers that they are.’* Assistant Vice President, Research Foundation (14^th^ November 2014)

Informants noted that the complexity of adopting innovations from the UK or other contexts ‘similar’ to the US is just as much as if they were from a low-income context. Health systems, regulatory barriers, scope-of-practice law, finance and professional boundaries make the transfer of innovation or learning as complex between rich countries as between poor countries. Some argued that, in some senses, there are more similarities between the UK and Ghana as there are between the UK and the US:

‘*…you could go further with this. You could make the argument that structurally, the Ghanian system is far closer to the UK system than the American system will ever be….it is centrally administered, district-based health system, much like the NHS is. And so theoretically, ideas from Ghana would be more relevant than ideas from the US. But that’s not how the learning works. And that’s the cultural arrogance piece of the postcolonial legacy that I think interferes.’* Vice President Innovation think tank (10^th^ October 2014)

If diffusion of innovation is as complex between high-income contexts as it is from low-to-high contexts, then it is as much of a curiosity that learning has tended to happen between the ‘usual suspects’ – high-income countries with geo-political or regional ties. The learning process, or diffusion of innovation from low-to-high income countries, whilst in theory probably no different in complexity to diffusion of innovations between high-income contexts, clearly possesses additional layers of complexity due to the challenges resulting in overcoming recipient sentiment regarding the source of the innovation. As one respondent puts it – "that’s not how the learning works". There are deeply embedded, prevalent and enduring assumptions that the global North teaches, and the global South, learns. The conflicting sentiments that the informants noted in using the term ‘Reverse Innovation’ is due to the term synthetically meaning two conflicting things simultaneously – the world is flat, but really it isn’t.

## Discussion

In recent years, the North–south model of development, rooted in post-colonial assistance, has been heralded as archaic [11]. Development has been called into question as an industry that is often self-serving [20–22] and failing to demonstrate significant change [23, 24]. Also, the global health landscape has changed dramatically. Power and influence is more diffuse with a proliferation of significant new actors [25, 26], and emerging economies continue to challenge established markets. There are many examples of impressive health innovations originating from low- or middle-income countries [10, 27–44]. These have the potential to disrupt health systems; indeed there are many reasons why the bloated healthcare economies of high-income countries could benefit from leaner innovations and out-of-the-box thinking.

Keeping pace with these changes, the ‘Reverse Innovation’ movement recognizes that knowledge, skills and learning can come from anywhere, and even flow from low to high-income countries. It is no surprise that ‘Reverse Innovation’ has come to be a buzzword heard in many innovation, management and healthcare circles. In 2012, a thematic series in Globalization and Health set out to explore and promote ‘Reverse Innovation’. In 2013, the Ivey International Centre for Health Innovation issued an open call to invite proposals for ‘Reverse Innovations’ that could address Canada’s health system challenges [45]. More recently, the International Partnership for Innovative Healthcare Delivery (IPIHD), recently renamed Innovations in Healthcare (IIH) formed out of a partnership between the World Economic Forum, Duke University, and McKinsey & Company, operates a ‘Reverse Innovation’ working group to address how successful innovations in healthcare delivery from low-income settings can be replicated in high-income settings. The Centre for Health Market Innovations (http://healthmarketinnovations.org) and the Institute for Global Health Innovation collate many examples of potentially adoptable innovations (http://www.imperial.ac.uk/centre-for-health-policy/our-work/innovation-research-/).

Don Berwick, former Administrator of the Centers for Medicare and Medicaid Services wrote about the inspiring ideas that Low-income countries held, saying “We may well find ourselves not the teachers we thought we were, but students of those who simply will not be stopped under circumstances that would have stopped us long ago” [31]. Although there is increasingly vocal recognition of the potential to learn from low-income countries, the most recent incarnation, as a process of ‘Reverse Innovation,’ as opposed to other more neutral terminology is steeped in persistent attitudes that learning ought really still go the other way.

It is curious that the ‘Reverse Innovation’ term has gained traction in healthcare circles even when its use connotes a very different process to that in the management literature, where it originated. Its use in the management literature is less loaded, because it describes a process that occurs within the same organizational boundaries of a Multinational Corporation, managing a strategic entry into new markets, and where ownership of the Intellectual Property of the product is clear. When the term ‘Reverse Innovation’ is used in the healthcare space, the actors involved are entirely different, often diffuse, often without direct delivery capacity, and often the ‘owner’ of the innovation may have no interest *per se* in exporting it. Furthermore, often the innovation may be a technology but it may also be a process, a service, a type of new cadre, or even simply a principle, with no specific intellectual property ownership from the perspective of the innovator context. The motivation for and the driver of the learning process are both also one-sided – all from the high-income context. Low-income country innovators are not explicitly selling or exporting their ideas to high-income countries.

Our study shows that although it may be a strategic lever to persuade actors in an organizational change process in high-income countries, the risk highlighted by respondents here in the US is that the ‘Reverse Innovation’ narrative may send negative signals to potential adopters and also to the innovator contexts. Association of Reverse Innovation with frugality risks potential adopters viewing the innovation as a poor alternative to existing practice. The availability and production of cheap products is not limited to low-income countries, and this type of thinking raises important questions about how people in the US determine the value of goods, and speaks more to the US psyche than those producing innovations themselves. Equally, lean innovations may have been developed in low-income countries from good leadership not from the necessity of poverty. The suggestion that these countries would not have been able to develop innovative solutions had the cost pressure not been present may be offensive. It is not known whether low-income country actors would themselves agree with this sentiment – the perspectives of actors in these settings where the Reverse Innovations are developed have been largely absent from the discussion [46]. Future research might draw on the social marketing literature to better understand how these terms evoke positive and negative connotations.

From the perspective of a high-income country, learning from a context that might be considered to be ‘on par’ with the US would be termed something somewhat less loaded such as diffusion of innovation, or bidirectional innovation. However, learning from a low-income context is has been given the term Reverse Innovation perhaps because it runs counter to the prevailing notion of where knowledge, experience or evidence is considered to generally derive from. This sentiment evokes the same sense of paradox, or even oxymoron, as the phrase ‘patient-centered care’ might [47] or ‘affirmative action’. If care were truly patient-centered it would be inappropriate to refer to individuals as patients - it is a term very much centered on the healthcare professional. ‘Affirmative action’ (the process) aims to redress an important inequity but ‘affirmative action’ (the term) simultaneously reinforces it. Reverse Innovation, similarly, is oxymoronic – as if to say ‘we like your idea but feel threatened that you came up with it – we’re going to pretend that we view you as peers.’ ‘Reverse Innovation’ clearly evokes different things to different people and in our research, the term has complex, multi-layered, sometimes positive, sometimes negative, connotations. Future research should study the impacts and repercussions of the term ‘Reverse Innovation’ within the innovator contexts. But also more research is needed to explore the views of and impact of Reverse Innovation (the term and the process) in the source, low-income country contexts, in particular to examine further the presence (or absence) of any sense of exploitation as noted in our findings. The study of Reverse Innovation, although of benefit to populations in high-income countries, requires also the voice of those in low- and middle-income countries. There is a need for a better understanding of the supply side for innovations, and how to develop the market of ideas from these contexts. Linking in with our notion of ‘cultural arrogance’, there is a need to understand whether actors in low- and middle-income countries are enabled to promote their solutions and innovations to the countries that have historically been the providers, not the beneficiaries, of assistance.

Until this research is available, it is probably sensible to use the term sparingly, that is, think carefully about whom it is used for and by, as it will evoke different things to different people.

Greenhalgh [48] has already called for terms such as ‘knowledge translation’ to be dropped because they constrain how the link between knowledge and practice is conceptualized. They propose that discourse analysis might be used to make explicit the process by which certain types and sources of knowledge become defined as ‘best evidence’ and that a much wider menu of metaphors to illustrate the non-linear, networked generation, circulation and sharing of ‘knowledge’ is needed. Terms such as knowledge sharing, diffusion of innovation, disruptive innovation and social innovation are agnostic with respect to the development and income status of the countries involved in the sharing process but also do little to accurately represent the translation, implementation and scaling of innovation. It is simplistic to describe it as either a linear (translation) or passive (diffusion) process. If the adoption, for want of a better word, of innovations from low- to high-income countries is indeed a different process to that between high-income countries, then a definitive taxonomy is needed to establish how best to describe it, when and to whom.

## Conclusion

We cannot step into the minds of our informants [49] but through this type of inquiry we can infer certain issues based on the narratives and experiences of our informants. The term ‘Reverse Innovation’ seems to attend to enduring sensibilities that knowledge that matters is ostensibly produced by high-income countries. However, at the same time, the use of the term may also have value as part of the sales pitch part of the process when it is perceived to be going the ‘other way’. The ‘cultural arrogance’ referred to by our informants speaks to a resistant, hegemonic perspective akin to hubris – excessive pride. Although it is the high-income country actors that are seeking out ideas for reform from contexts that traditionally were considered the weaker, fragile and resource-poor contexts, ‘Reverse Innovation’ mitigates the bruising of collective pride that such a process might inflict. To contrast, why not just call it ‘learning’, for example?

## Abbreviations

GE, general electric; IRB, Institutional Review Board; NHS, UK National Health Service; UK, United Kingdom; US, United States.

## Additional file

Additional file 1: Definitions of selected terms related to different types of innovation. (DOC 30 kb)
