# Supplementary material for: That’s not how the learning works – the paradox of Reverse Innovation: a qualitative study
Source: Global Health. 2016 Jul 5;12:36. doi: 10.1186/s12992-016-0175-7 (PMC4932777; doi:10.1186/s12992-016-0175-7)
Supplement: Additional file 1: — Definitions of selected terms related to different types of innovation. (DOC 30 kb) [file 12992_2016_175_MOESM1_ESM.doc]

Additional file 1: Definitions of selected terms related to different types of innovation

| Term | Year | Definition |
| --- | --- | --- |
| Frugal innovation | 2010 | Also referred to as “frugal engineering”, a type of innovation described as rethinking processes and models, doing more, with less, for many [1] |
| Disruptive innovation | 2003 | A process by which a product or service takes root initially in simple applications at the bottom of a market and then relentlessly moves up market, eventually displacing established competitors.[2] This process builds on the concept of ‘disruptive technologies’ which are technologies that underperform established products in mainstream markets, but bring to the market other value propositions than have been available previously (for example, they are typically cheaper, simpler, smaller, and, frequently, more convenient to use. |
| Jugaad | 2011 | A colloquial Hindi word for grassroots innovation that can mean “innovative fix”, sometimes of poor quality or of unaccepted standards used for solutions that bend rules. [5] |
| Innovation blowback | 2005 | Described as a wave of disruptive product and process innovations arising from emerging markets that compete for market share in Europe and the United States and require western companies to urgently reposition themselves to deal with these offshore challenges.[3] |
| Bottom of pyramid | 2004 | The socio-economic theory that new business opportunities lie in designing and distributing goods and services for poor communities, those at the ‘bottom of the pyramid’.[4] |

[1] Bhatti,Y. Khilji, S. & Basu, R. 2013. “Frugal Innovation” in Globalization, Change and Learning in South Asia. Edited by Rowley, Chris & Khilji, Shaista. UK: Chandos Publishing.

[2] Christensen C. The Innovator’s Dilemma: When New Technologies Cause Great Firms to Fail

[3] John Seely Brown and John Hagel III "Innovation blowback: Disruptive management practices from Asia (McKinsey Quarterly)" (2005)

[4] Prahalad 2004 The Fortune at the Bottom of the Pyramid

[5] Pushyamitra J, Anirudha A, & V Manish, Grass root creation to organized innovation: A role of Jugaad in organized industry. Strategies & Innovation for Sustainable Organizations Conference, March 2011. Macmillan.
